# New Potential Biomarker for Methasterone Misuse in Human Urine by Liquid Chromatography Quadrupole Time of Flight Mass Spectrometry

**DOI:** 10.3390/ijms17101628

**Published:** 2016-09-24

**Authors:** Jianli Zhang, Jianghai Lu, Yun Wu, Xiaobing Wang, Youxuan Xu, Yinong Zhang, Yan Wang

**Affiliations:** 1 State Key Laboratory of Bioactive Substance and Function of Natural Medicines, Institute of Materia Medica, Chinese Academy of Medical Sciences & Peking Union Medical College, Beijing 100050, China; zhangjianli@chinada.cn; 2 China Anti-Doping Agency, Beijing 100029, China; wuyun@chinada.cn (Y.W.); wangxiaobing@chinada.cn (X.W.); xuyouxuan@chinada.cn (Y.X.); zhangyinong@chinada.cn (Y.Z.)

**Keywords:** methasterone, new biomarker, human urine, doping control, liquid chromatography time-of-flight tandem mass spectrometry

## Abstract

In this study, methasterone urinary metabolic profiles were investigated by liquid chromatography quadrupole time of flight mass spectrometry (LC-QTOF-MS) in full scan and targeted MS/MS modes with accurate mass measurement. A healthy male volunteer was asked to take the drug and liquid–liquid extraction was employed to process urine samples. Chromatographic peaks for potential metabolites were hunted out with the theoretical [M − H]^−^ as a target ion in a full scan experiment and actual deprotonated ions were studied in targeted MS/MS experiment. Fifteen metabolites including two new sulfates (S1 and S2), three glucuronide conjugates (G2, G6 and G7), and three free metabolites (M2, M4 and M6) were detected for methasterone. Three metabolites involving G4, G5 and M5 were obtained for the first time in human urine samples. Owing to the absence of helpful fragments to elucidate the steroid ring structure of methasterone phase II metabolites, gas chromatography mass spectrometry (GC-MS) was employed to obtain structural information of the trimethylsilylated phase I metabolite released after enzymatic hydrolysis and the potential structure was inferred using a combined MS method. Metabolite detection times were also analyzed and G2 (18-nor-17β-hydroxymethyl-2α, 17α-dimethyl-androst-13-en-3α-ol-ξ-*O*-glucuronide) was thought to be new potential biomarker for methasterone misuse which can be detected up to 10 days.

## 1. Introduction

Methasterone (superdrol, and methyldrostanolone) is an orally active anabolic agent exhibiting androgenic activity [1,2]. As 17α-methylation makes a valuable contribution to the enhancement of anabolic effect and considerably increases the anabolic strength of the steroid by heightening its resistance to metabolism in skeletal muscle tissue [2,3], methasterone shares many characteristic of its non-17α-alkylated counterpart drostanolone, a substance with high anabolic activity. Methasterone is also prone to be contaminated in nutritional supplements [4], and athletes may favor methasterone for its moderate anabolic properties and fat burning ability. Thus, its use in- and out-of-competition is prohibited by the World Anti-Doping Agency (WADA) [5]. As for its damage to athletes, it is noted that the addition of a methyl group at carbon 17-α can delay methasterone eliminating from body and increase hepatic toxicity [6].

Owing to the lack of an ionizable moiety, methasterone metabolism has been investigated mainly by gas chromatography mass spectrometry (GC-MS) [7,8,9]. Human liver microsomes and an uPA^+/+^-SCID chimeric mouse model were employed to produce its different metabolites, and the reduction in C3 together with hydroxylation in C2, C12 (minor), C16 and C20 constitute the main metabolism pathways [10,11,12]. Methasterone and its hydroxylated metabolites were also excreted as glucuronidated compounds, while no sulfated metabolites could be detected in phase II metabolism [13]. Up to date, methasterone and its main metabolite (dihydromethasterone) in free and glucuronide fractions were detected in human urine by GC-MS after taking the nutritional supplement containing 10 mg methasterone and the amount of dihydromethasterone was five times greater than that of parent drug [14]. Based on our experiences, two biomarkers, parent drug and its C3-reduced metabolite, were employed for monitoring methasterone misuse and an enzymatic hydrolysis step was involved prior to GC-MS analysis in doping control field, which was time-consuming and indirect [15]. Up to now, the application of LC-MS/MS has been increasingly used to identify phase II metabolites [13,16,17]. The objective of this paper is to identify and characterize intact methasterone metabolites (mainly sulfate and glucuronide conjugates) by liquid chromatography coupled with quadrupole time-of-flight mass spectrometric technique (LC-QTOF-MS) and gas chromatography mass spectrometry and find biomarkers to improve our routine analysis in doping control.

## 2. Results

### Proposed Metabolic Pathway and Application for Doping Control

**Figure 1:**
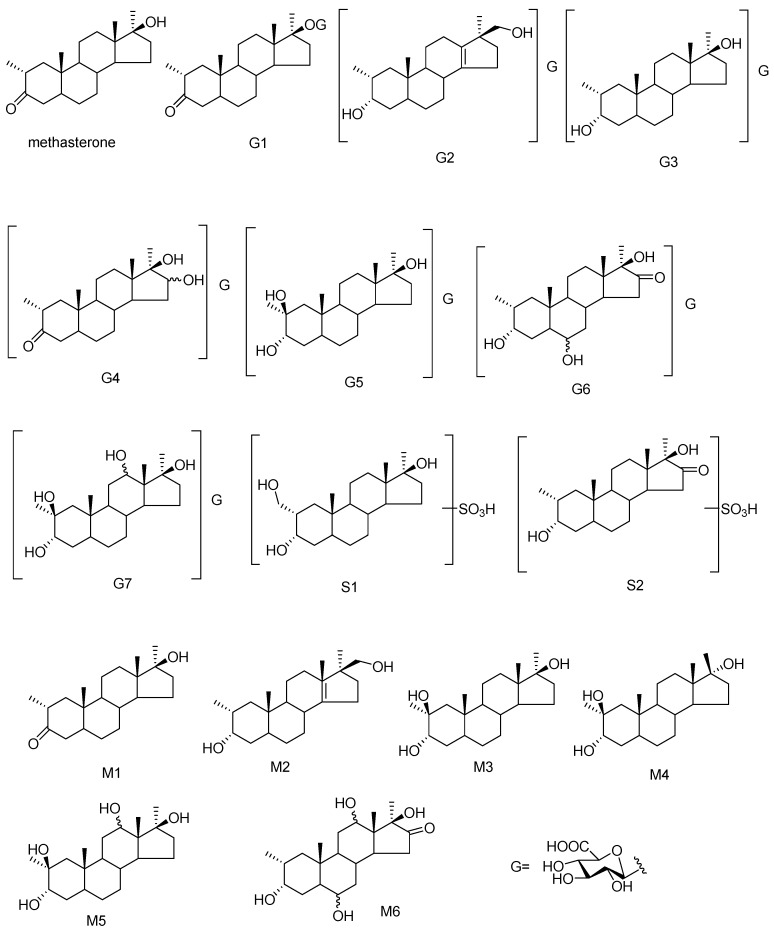
The chemical structures of methasterone and its intact metabolites (G2, G6, G7, S1, S2, M2, M4 and M6 were unreported metabolites, and G4, G5 and M5 were obtained for the first time in human urine).

**Figure 2:**
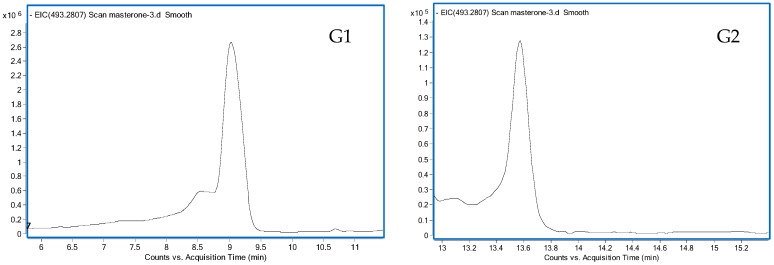

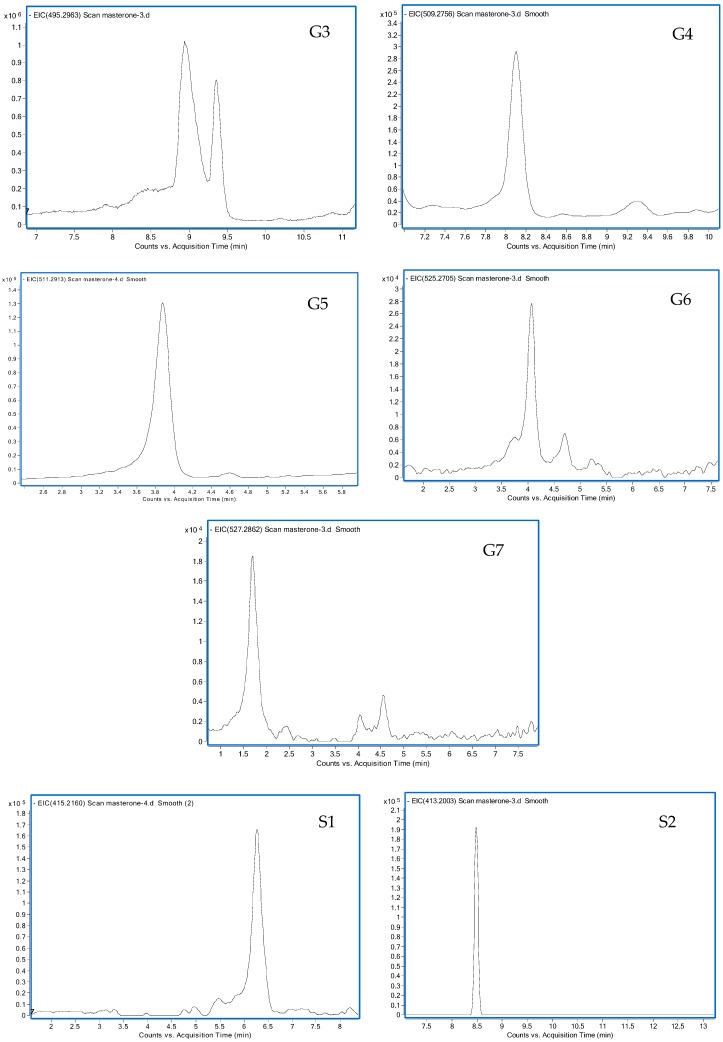

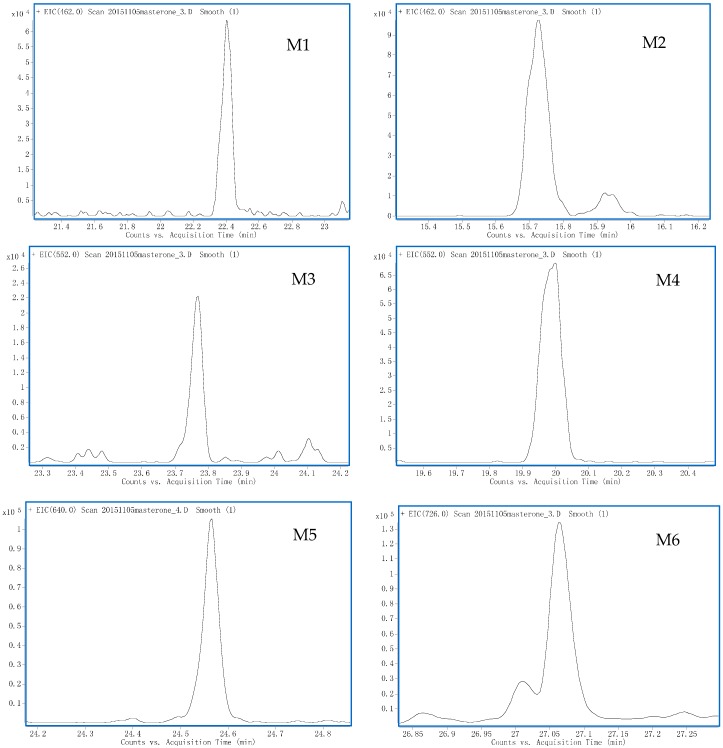
EIC chromatograms of 15 metabolites (EIC chromatograms of G1, G2, G3, G4, G5, G6, G7, S1 and S2 were required by LC-QTOF-MS in negative mode, while EIC chromatograms of M1, M2, M3, M4, M5 and M6 were required by GC-MS).

The chemical structures of methasterone and its 15 metabolites are shown in Figure 1. The major metabolic reaction of methasterone focused on hydroxylation at C2, C6, C12 and C16 in steroidal skeleton and glucuronidation, while sulfation constituted a minor metabolic pathway and only two sulfate conjugates were isolated. Free and glucuronide-conjugated metabolites were the major metabolite classes and 15 metabolites in total were detected. Four pairs of excreted metabolites contained free and conjugated fractions, which included M1 and G1, M2 and G2, M3 and G5, and M5 and G7. One dihydroxylated (M5) and one trihydroxylated (M6) metabolites were also detected in the free fraction, which was advantageous to facilitate their elimination from human body. The extract ion chromatography (EIC) chromatograms of 15 metabolites are shown in Figure 2.

**Figure 3:**
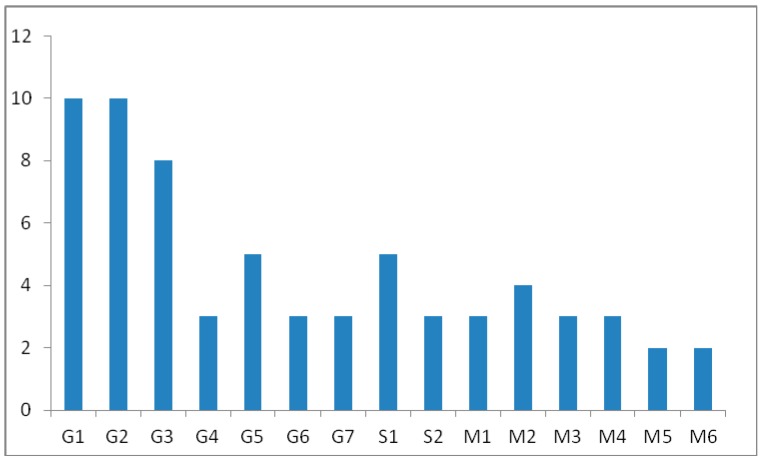
Detection times (days) of the different metabolites monitored in the administration study urine samples collected after oral administration.

In order to estimate the utility of all metabolites to prolong and/or improve the detection of methasterone misuse, the detection windows of all metabolites described above were appraised in administration study urine samples (Figure 3). Detection times commonly ranged from three days to approximately 10 days. Only two of them were considered important as the most abundant and long term detectable. G1 and G2 clearly extend the detection window up to 10 days indicating the longest retrospectivity for the detection of methasterone misuse. It was also noted that the detection times of phase II metabolites were longer than those of phase I metabolites.

## 3. Discussion

### 3.1. Identification of Potential Glucuronide-Conjugated Metabolites

As for glucuronide conjugates, [M + NH_4_]^+^ and [M − H]^−^ were shown in full-scan positive and negative modes. Targeted MS/MS analysis indicated product ions at *m*/*z* 141,159, and 177 in positive mode together with *m*/*z* 75, 85, 113, and 175 in negative mode [18,19,20] were generated and no detailed information about steroidal ring was presented. Removal of the glucuronic group by enzymatic hydrolysis was essential here for GC-MS analysis. Identification of the phase I metabolites was obtained through GC-MS analysis of TMS ethers and TMS enol-TMS ethers. Mass spectrum data and the proposed mass spectrometry fragmentation pathway of the newly reported metabolites are presented below.

#### 3.1.1. G1 and G2

**Figure 4:**
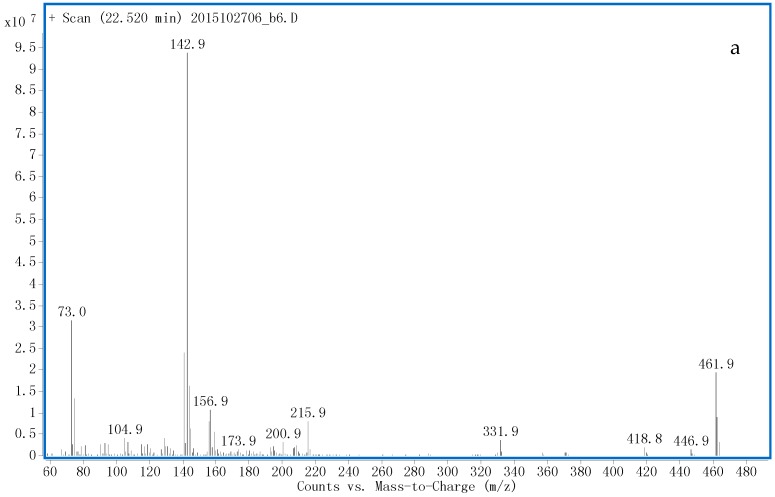

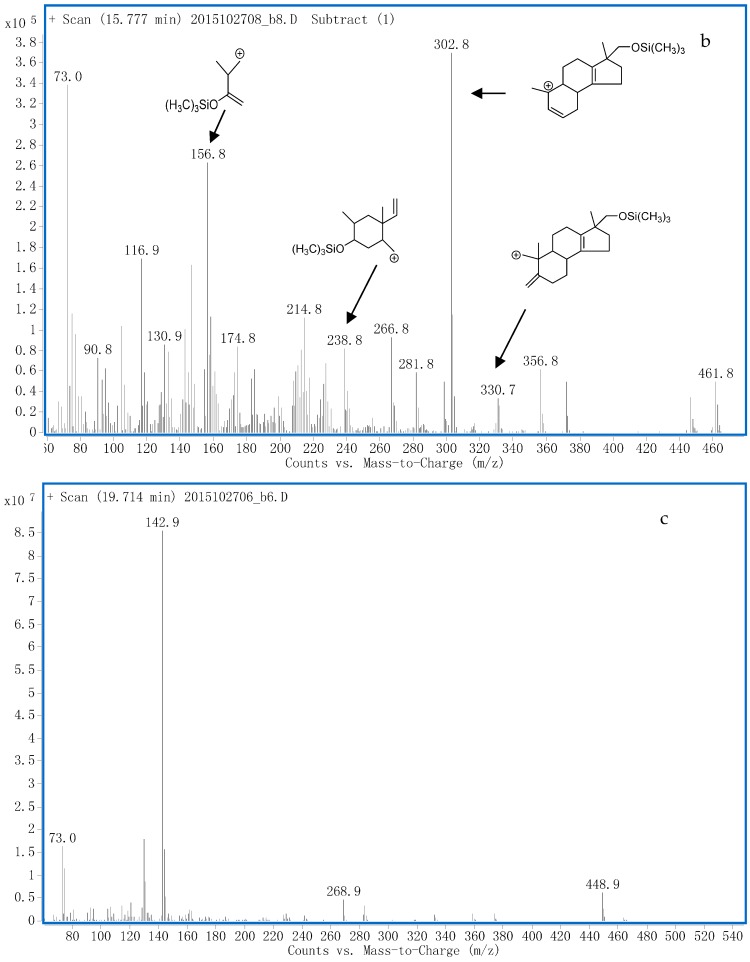

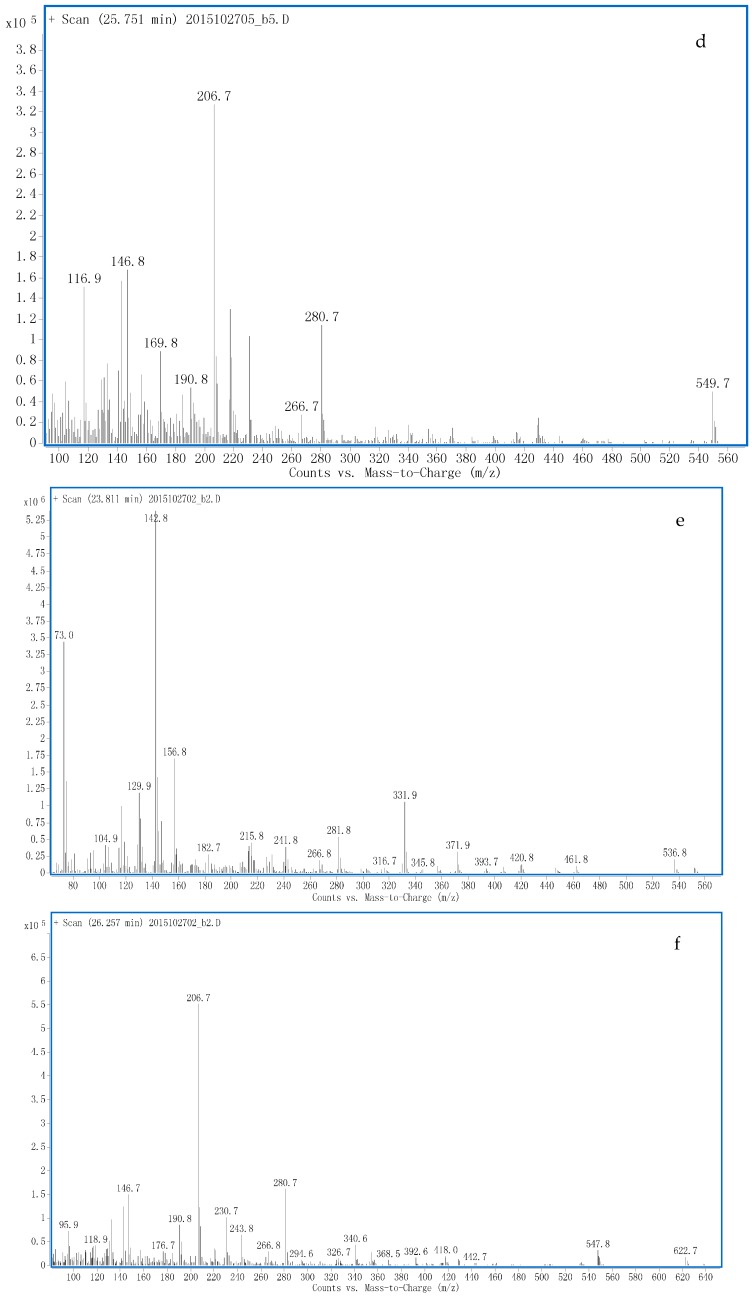

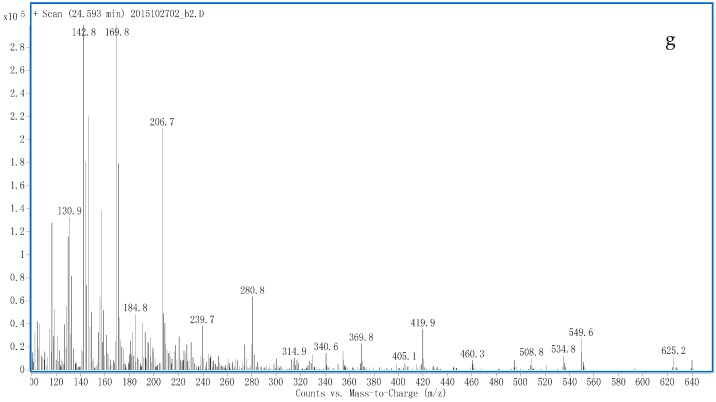
The electron ionization mass spectra of TMS derivatives of the phase II metabolites of: G1 (**a**); G2 (**b**); G3 (**c**); G4 (**d**); G5 (**e**); G6 (**f**); and G7 (**g**) released after hydrolysis.

Two peaks of [M − H]^−^ at *m*/*z* 493.2807 with RT 9.2 (G1) and 13.6 (G2) min were detected using LC-QTOF-MS analysis (Figure S1). This *m*/*z* implied the same chemical composition as unchanged methasterone. The LC-fraction containing G1 was subjected to enzymatic hydrolysis and TMS derivatization, a peak with RT 22.45 min was generated after GC-MS analysis and the electron ionization (EI) mass spectrum (Figure 4a) was identical to unchanged methasterone. As for G2, the EI mass spectrum (Figure 4b) generated characteristic ions at *m*/*z* 461.8 (M), 446.8 (M − CH_3_), 371.8 (M − TMSOH), 356.8 (M − CH_3_ − TMSOH), 281.8 (M − 2TMSOH) and 266.8 (M − CH_3_ − 2TMSOH), the fragments at *m*/*z* 302.8 and 156.8 were suggested to originate from A-ring cleavage and no the *m*/*z* 143 ion corresponding to 17-methyl-17-ol group was presented, which supported the presence of 18-CH_3_ rearrangement [21,22,23]. Thus, G1 and G2 were proposed as methasterone-17β-*O*-glucuronide and 18-nor-17β-hydroxymethyl-2α, 17α-dimethyl-androst-13-en-3α-ol-ξ-*O*-glucuronide, respectively. G2 was an unreported metabolite, which could be detected up to 10 days after administration.

#### 3.1.2. G3

One peak of [M−H]^−^ at *m*/*z* 495.2972 with RT 9.4 (G3) was detected using LC-QTOF-MS analysis (Figure S2), which suggested one reduction in the steroidal ring. The LC-fraction containing G3 was subjected to enzymatic hydrolysis and TMS derivatization, a peak with RT 19.72 min was presented after GC-MS analysis and the electron ionization (EI) mass spectrum (Figure 4c) was identical to 2α, 17α-dimethyl-5α-androstane-3α, 17β-diol [15]. Thus, G3 was identified as 2α, 17α-dimethyl-5α-androstane-3α, 17β-diol-ξ-*O*-glucuronide.

#### 3.1.3. G4

A peak of [M − H]^−^ at *m*/*z* 509.2765 with RT 8.1 (G4) min was detected using LC-QTOF-MS analysis (Figure S3), which implied one hydroxylation. The LC-fraction containing G4 was subjected to enzymatic hydrolysis and TMS derivatization, a peak with RT 25.8 min was yielded after GC-MS analysis and the characteristic ions at *m*/*z* 217.8 and 230.8 were present in the EI mass spectrum of tris-TMS derivatives of the phase I metabolites of G4 released after hydrolysis, which supported the presence of 16-hydroxylated steroids (Figure 4d) [24]. Thus, G4 were proposed as 2α, 17α-dimethyl-5α-androstane-16ξ, 17β-diol-3-One-ξ-*O*-glucuronide, which was detected for the first time in human urine.

#### 3.1.4. G5

A peak of [M − H]^−^ at *m*/*z* 511.2913 with RT 3.9 min was detected using LC-QTOF-MS analysis (Figure S4), which implied one hydroxylation and one reduction. The LC-fraction containing G5 was subjected to enzymatic hydrolysis and TMS derivatization. The EI mass spectrum (Figure 4e) was in agreement with 2α, 17α-dimethyl-5α-androstane-2β, 3α, 17β-triol [9]. Thus, G5 was established as 2α, 17α-dimethyl-5α-androstane-2β, 3α, 17β-triol-ξ-*O*-glucuronide, which was detected for the first time in human urine.

#### 3.1.5. G6

A peak of [M − H]^−^ at *m*/*z* 525.2712 with RT 4.0 min was detected using LC-QTOF-MS analysis (Figure S5), which implied two hydroxylations. The LC-fraction containing G6 was subjected to enzymatic hydrolysis and TMS derivatization. In GC-MS analysis, the characteristic ions at 243.8 (Figure 4f) showed the presence of 16-keto group and 3-keto group was accordingly reduced [24]. Another hydroxyl-group was unknown, and yet it was obvious that it was not linked to C12 and C2 (lack of *m*/*z* 170, 157) [9,24], but rather at C6 [1]. Thus, G6 was tentatively proposed as 2α, 17α-dimethyl-5α-androstan-3α, 6ξ, 17β-triol-16-one-ξ-*O*-glucuronide, which was an unreported metabolite.

#### 3.1.6. G7

One peak of [M − H]^−^ at *m*/*z* 527.2866 with RT 1.7 min was detected using LC-QTOF-MS analysis (Figure S6), which implied two hydroxylations and one reduction. The LC-fraction containing G7 was subjected to enzymatic hydrolysis and TMS derivatization. The EI mass spectrum (Figure 4g) yielded the characteristic ions at *m*/*z* 640.3, 549.6, 460.3, 369.8 and 280.8 (sequential losses of HOTMS), 419.9, 169.8 and 142.8. The fragment ion at *m*/*z* 419.9 and 169.8 were typical for the presence of 2-and 12-hydroxylated steroids [9,10,24]. Thus, G7 was tentatively proposed as 2α, 17α-dimethyl-5α-androstan-2β, 3α, 12ξ, 17β-tetrol-ξ-*O*-glucuronide, which was an unreported metabolite.

### 3.2. Identification of Potential Sulfate-Conjugated Metabolites

Two new sulfate conjugates were found through weak acidic extraction (pH 5), product ion analysis indicated the presence of two characteristic ions at *m*/*z* 97 and 80 [25] relative to sulfonic acid and no detailed steroidal skeleton information was produced. Thus, elimination of the sulfate group was necessary here for GC-MS analysis, and elucidation of the phase II metabolites was obtained by GC-MS analysis of TMS ethers and TMS enol-TMS ethers, and comparison of mass spectral data with reference data from literature and standards. The mass spectra and the deduced mass spectrometric fragmentation pathway of these metabolites are demonstrated and discussed below.

#### 3.2.1. S1

**Figure 5:**
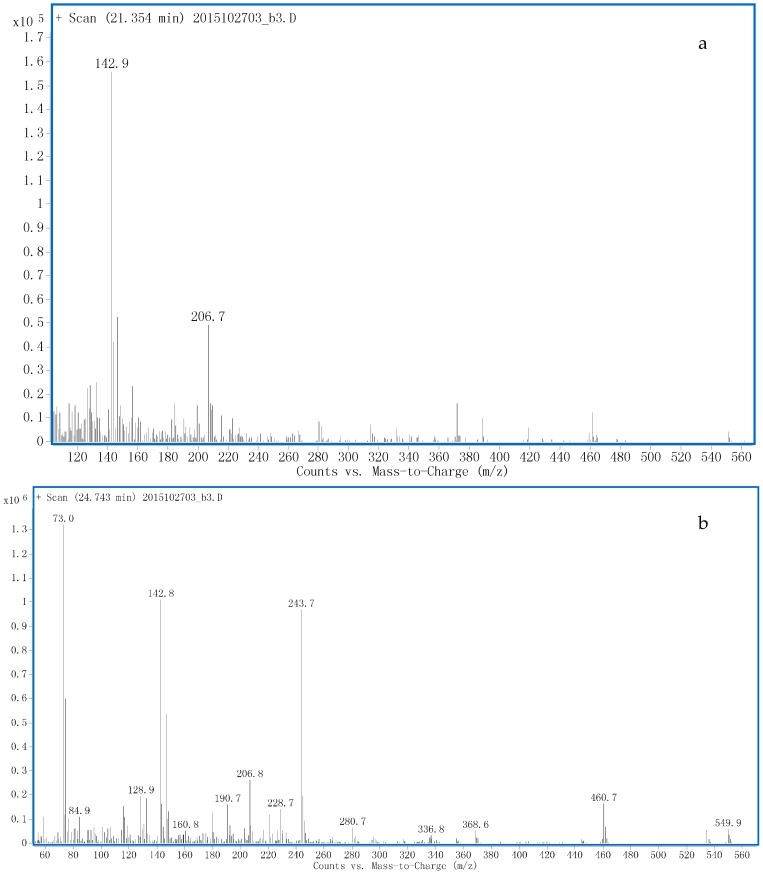
The Electron ionization mass spectra of TMS derivatives of the phase II metabolites of: S1 (**a**); and S2 (**b**) released after hydrolysis.

One peak of [M − H]^−^ at *m*/*z* 415.2168 (S1) with RT 6.3 min was detected using LC-QTOF-MS analysis (Figure S7), which implied a hydroxylation and a reduction (of the 3-keto group). The LC-fraction containing S1 was subjected to enzymatic hydrolysis and TMS derivatization. The characteristic ions at *m*/*z* 551.7, 461.7, 371.9 (sequential losses of HOTMS), 157.1, 142.9 were observed in EI mass spectrum (Figure 5a), which was identical to 2α-hydroxymethyl-17α-methyl-5α-androstan-3α, 17β-diol [9]. Thus, S1 was proposed as 2α-hydroxymethyl-17α-methyl-5α-androstan-3α, 17β-triol-ξ-*O*-sulfate.

#### 3.2.2. S2

One peak of [M − H]^−^ at *m*/*z* 413.2006 with RT 8.5 min was detected using LC-QTOF-MS analysis (Figure S8), which implied a hydroxylation. The LC-fraction containing S2 was subjected to enzymatic hydrolysis and TMS derivatization. In GC-MS analysis, the fragment ion at *m*/*z* 243.7 was present in the EI mass spectrum (Figure 5b) supporting the presence of a 16-keto group and 3-keto group was reduced accordingly. Thus, S2 was established as 2α, 17α-dimethyl-5α-androstan-3α, 17β-diol-16-one-ξ-*O*-sulfate.

### 3.3. Identification of Potential Free Metabolites

Owing to lack of an ionizable moiety, hunting down methasterone unconjugated excreted metabolite mainly relied on GC-MS instrumentation with full-scan mode. Six free urinary metabolites were detected, which included M1 and M2 (free fractions of G1 and G2), M3 (free fraction of G5), M4 (no conjugated fraction), M5 (free fraction of G7), and M6 (trihydroxylated metabolite). No dihydrogenated metabolite (free fraction of G3) was detected, which was previously reported as the main metabolite. M2, M4 and M6 were unreported metabolites and M4 together with M6 were excreted only in the free fraction. The structural elucidation of M4 and M6 is presented as follows.

#### 3.3.1. M4

**Figure 6:**
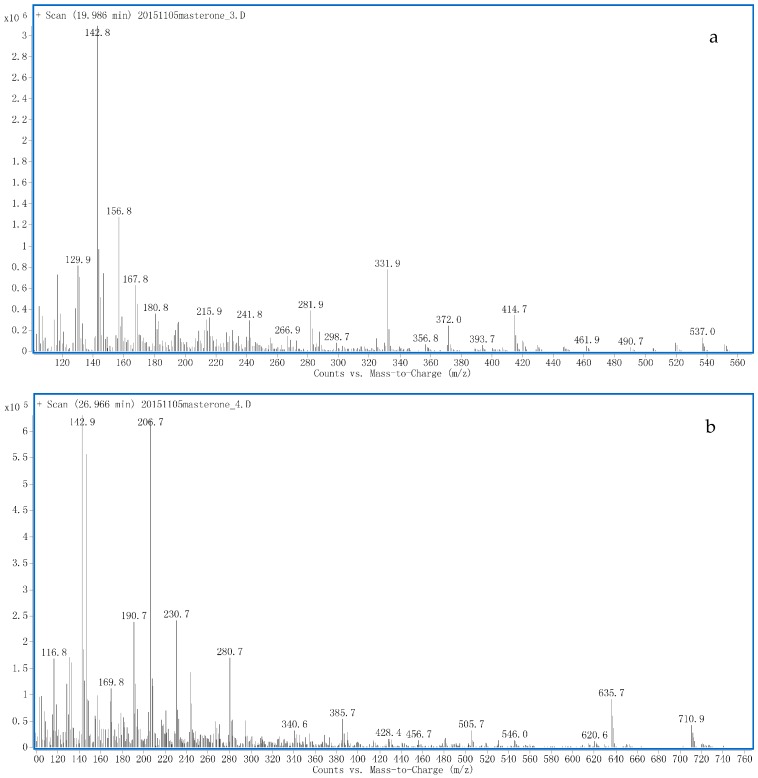
The Electron ionization mass spectra of TMS derivatives of: M4 (**a**); and M6 (**b**).

M4 was isolated in the free fraction and the EI mass spectrum (Figure 6a) exhibited a molecular ion at *m*/*z* 552 (tri-TMS derivatives) with RT 20 min, which implied a hydroxylation and a reduction. The fragment ions at *m*/*z* 537, 461.9, 420, 373, 331.9, 281.9, 156.8 and 142.8 were identical to M3 [9], but their RTs were different. Thus, M4 was tentatively proposed as 2α, 17β-dimethyl-5α-androstan-2β, 3α, 17α-triol, which was an unreported metabolite in human urine.

#### 3.3.2. M6

A peak with RT 27.0 min was obtained by the extraction of the *m*/*z* 726 suggesting potential trihydroxylation. GC-MS analysis (Figure 6b) showed the fragment ions at *m*/*z* 710.9 (M − CH_3_), 635.7, 546.0, 456.0 (sequential losses of HOTMS), 243.8 (corresponding to a 16-keto group), 190.7 (relative to C6-hydroxylation), 169.8 (corresponding to C12-hydroxylation) and 142.8 [10,24]. C3-keto group was accordingly reduced. Thus, M6 was tentatively proposed as 2α, 17α-dimethyl-5α-androstan-3α, 6ξ, 12ξ, 17β-tetrol-16-one, which was an unreported metabolite.

## 4. Materials and Methods

### 4.1. Chemicals and Reagents

Methasterone was donated from the Doping Control Laboratory of Madrid, State Anti-Doping Agency (Madrid, Spain). All HPLC grade solvents were purchased from Dima Tech. Inc. (Richmond Hill, CA, USA). Sodium dihydrogen phosphate monohydrate and disodium hydrogen phosphate dehydrate were obtained from Sinopharm Chemical Reagent Co., Ltd. (Beijing, China). Sulfatase from Helix pomatia–Type H-2 was purchased from Sigma-Aldrich (Saint Louis, MO, USA).

### 4.2. Instrumentation

#### 4.2.1. LC/QTOF-MS Studies

An Agilent 1290 Series LC system (Waldbronn, Germany) was employed for the chromatographic separation, which was made up of a vacuum degasser, a high-pressure binary pump, an autosampler with a sample tray and a column oven. The LC were installed with an Agilent Zorbax XDB-C18 column (50 mm length, 2.1 mm inner diameter, 3.5 µm particle size) connected to a filter (particle size 0.5 µm), and the separation was conducted at constant temperature (40 °C). The mobile phase contained water (eluent A: 10 mM HCOONH_4_, 0.05% HCOOH for positive ion scan, and 5 mM CH_3_COONH_4_ for negative ion scan) and acetonitrile (eluent B). A gradient was used beginning at 10% B and increasing to 90% B in 15 min, 100% B at 17 min (total run time: 17 min). The column was finally re-equilibrated at 10% B for 5 min. The flow rate was fixed at 0.3 mL/min.

The mass spectrometer was composed of an orthogonal acceleration quadrupole time-of-flight mass spectrometer (6538 Accurate- Mass QTOF LC/MS; Agilent Technologies, Santa Clara, CA, USA) installed with an orthogonal electrospray ionization (ESI) source, a temperature-stabilized analogue-to-digital converter (ADC) handled at 2 GHz and a microchannel plate (MCP) handled at 725 V. Ionization took effect in the positive and negative experiments and nitrogen was employed as the drying and nebulizing gas. The drying gas flow rate and temperature were fixed at 10 L/min and 350 °C, respectively, and the nebulizer gas pressure was set at 35 psi. The capillary voltage was set at 5500 V. The fragmentor voltage was 130 V. Full scan mass spectral data were obtained from *m*/*z* 50 to 1100 at a rate of 1.5 scan/s while targeted MS/MS data were acquired from *m*/*z* 60 to 600 at the same rate. The other QTOF-MS parameters (transfer optic and ion focus voltages, quadrupole lens, TOF and detector voltages) were automatically optimized through the instrument auto-tuning step, performed daily. The mass calibration was employed in the period of the analysis to obtain the preferred mass accuracy, by continuously injecting two references (hexakis (1H, 1H, 3H-tetrafluoropropoxy) phosphazine and purine; Agilent Technologies, Santa Clara, CA, USA) into the ESI source from a second orthogonal nebulizer with the samples at the same time.

#### 4.2.2. GC-MS Analysis

In GC-MS analysis, an Agilent 7890 GC was installed with a quadrupole 5975 mass spectrometer (Agilent Technologies, Wilmington, DE, USA) and a HP-1 cross-linked capillary column (0.2 mm i.d., 25 m length, 0.11 µm coating thickness, J&W scientific column from Agilent Technologies, Wilmington, DE, USA). Injections were automatically conducted in split mode (10:1) at 280 °C. The initial column temperature was set at 177 °C and then increased at a rate of 3 °C/min to 245 °C and finally 17 °C/min to 320 °C (holding 2.5 min). The total run time was fixed at 29.58 min. The transfer line temperature was maintained at 280 °C, and the flow rate of high purity helium (carrier gas) was set at 0.60 mL/min. The electron ionization (EI) mass spectrometer was operated under the following conditions: electron source temperature, 230 °C; electron energy, 70 eV. Data were obtained in the scan range of *m*/*z* 40–700.

### 4.3. Sample Preparation

#### 4.3.1. Unconjugated Steroids

Six milliliters of urine was buffered to pH 11.0 with 2 mL of an aqueous solution containing potassium carbonate and potassium bicarbonate (20%, 1:1, *w*/*w*) and then extracted with 4 mL of tert-butyl methyl ether (TBME), the mixture vortexes for 0.5 min, and then centrifuged for 5 min. The organic layer was transferred to a new glass tube, blown to dryness at 60 °C for 40 min, and then the dry residues were derivatized with 50 μL of a mixture of MSTFA/NH_4_I/2-mercaptoethanol (1000:2:6, *v*/*w*/*v*) and incubated at 60 °C for 30 min. The derivatized extracts were placed to injection vials and 1 μL was analyzed by GC-MS.

#### 4.3.2. Glucuronide and Sulfate-Conjugated Steroids

The remaining aqueous portion was then adjusted to pH 5.0 and extracted with 5 mL ethyl acetate. The mixture was vortexed for 0.5 min, and then the organic layer was transferred to a fresh glass tube, blown to dryness at 60 °C, and the dry residue dissolved in 200 μL of a mixture of pure water containing 20 mM ammonium acetate together with methanol (50:50, *v*/*v*), and 20 μL of the reconstituted solution was injected for LC-MS/MS analysis.

### 4.4. LC Fractionation and Enzymatic Hydrolysis

A LC fractionation of urine sample was performed in order to separate these metabolites, and sample processing mentioned above in “Section 4.3” was introduced to the urine aliquot including the most abundant composition of sulfate-conjugated metabolite (S1). The organic phases of extracts were combined, blown to dryness, reconstituted in an injection vial. Fractionations were manually collected according to the expected retention time (RT ± 0.2 min) of the metabolite peaks. A enzymatic hydrolysis was conducted as follows: fractions were processed again with 2 mL acetic acid/sodium acetate buffer (0.2 M, 6.8/43.2 (*v*/*v*), pH 5.5) by adding 50 μL of sulfatase from Helix pomatia and incubated at 55 °C for 2 h, and then extracted with 5 mL of TBME. After centrifugation, the organic layer was transferred and blown to dryness. The dry residues were derivatized with 50 μL of a mixture of MSTFA/NH_4_I/2-mercaptoethanol (1000:2:6, *v*/*w*/*v*), incubated at 60 °C for 30 min and then transferred to injection vials. One microliter of the derivatized extracts was analyzed by GC-MS.

### 4.5. Excretion Studies

Forty-milligram dosage of methasterone (Yuancheng Bio-Pharm Co., Ltd. Wuhan, China) was taken orally by a healthy male volunteer (44 year, 65 kg). The volunteer was asked to sign informed consent prior to the metabolic studies and the clinical project was approved by the ethical committee of China Anti-Doping Agency on 15 May 2015 (Identification code: 201502). Urine blank was collected before administration, and the urine samples were collected after administration at the following time periods: all urine samples within 0–48 h were collected and morning urines were collected daily from Day 3 to Day 40 after administration. The urine samples were stored at −20 °C.

## 5. Conclusions

In this study, the metabolic profile of methasterone was investigated by means of LC coupled to hybrid QTOF-MS instrumentation. Fifteen metabolites including seven glucuronide-conjugated and six free metabolites together with two sulfate-conjugated ones were found for methasterone. The nine phase II metabolites were detected with accurate mass measurement by LC-QTOF-MS instrumentation, and a LC cleanup procedure was employed to isolate these metabolites and the LC-fraction containing relative conjugated metabolites was subjected to enzymatic hydrolysis and TMS derivatization. The detailed structural information in steroidal skeleton was based on GC-MS analysis. G4, G5 and M5 were reported for the first time in human urine, and eight metabolites including G2, G6, G7, S1, S2, M2, M4 and M6 were unreported metabolites both in vivo and in vitro. The conjugated metabolite G2 was considered as the novel potential biomarker for methasterone misuse in human urine for doping control. In our routine analysis, M1 and the free fraction of G3 are analyzed by GC-MS, which can be detected for three days and five days in urine samples after a single dose of 40 mg methasterone for oral administration by a healthy male volunteer, respectively. Due to the efficiency of enzymatic hydrolysis and derivatization, the direct detection of the reported glucuronide-conjugated metabolites in this study can obviously extend the detection window of methasterone, G2 as a new potential biomarker for methasterone misuse can be detected up to 10 days (Figure 3). To better support these results, synthesis of these metabolites should be performed for unambiguous characterization of their chemical structures.

## Supplementary Materials

Supplementary materials can be found at www.mdpi.com/1422-0067/17/10/1628/s1.
