# Supplementary material for: New Potential Biomarker for Methasterone Misuse in Human Urine by Liquid Chromatography Quadrupole Time of Flight Mass Spectrometry
Source: Int J Mol Sci. 2016 Sep 24;17(10):1628. doi: 10.3390/ijms17101628 (PMC5085661; doi:10.3390/ijms17101628)
Supplement: Supplementary file 1 [file ijms-17-01628-s001.pdf]

Supplementary Materials: New Potential Biomarker for Methasterone Misuse in Human Urine by Liquid Chromatography Quadrupole Time of Flight Mass Spectrometry

Jianli Zhang, Jianghai Lu, Yun Wu, Xiaobing Wang, Youxuan Xu, Yinong Zhang and Yan Wang

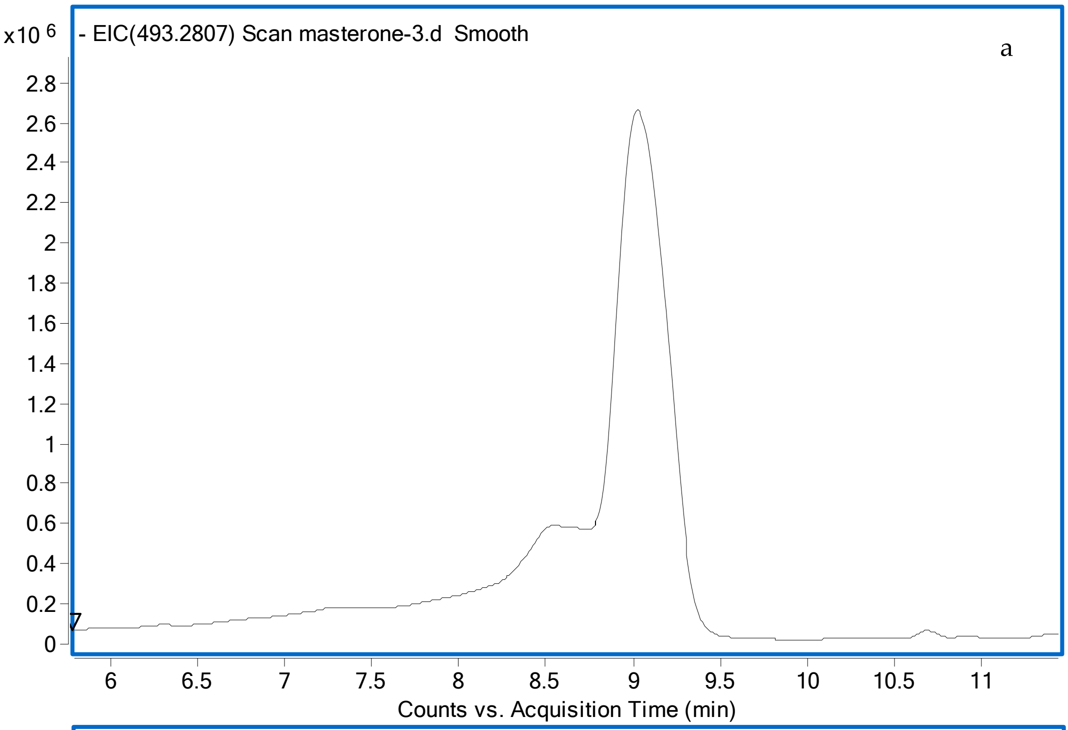

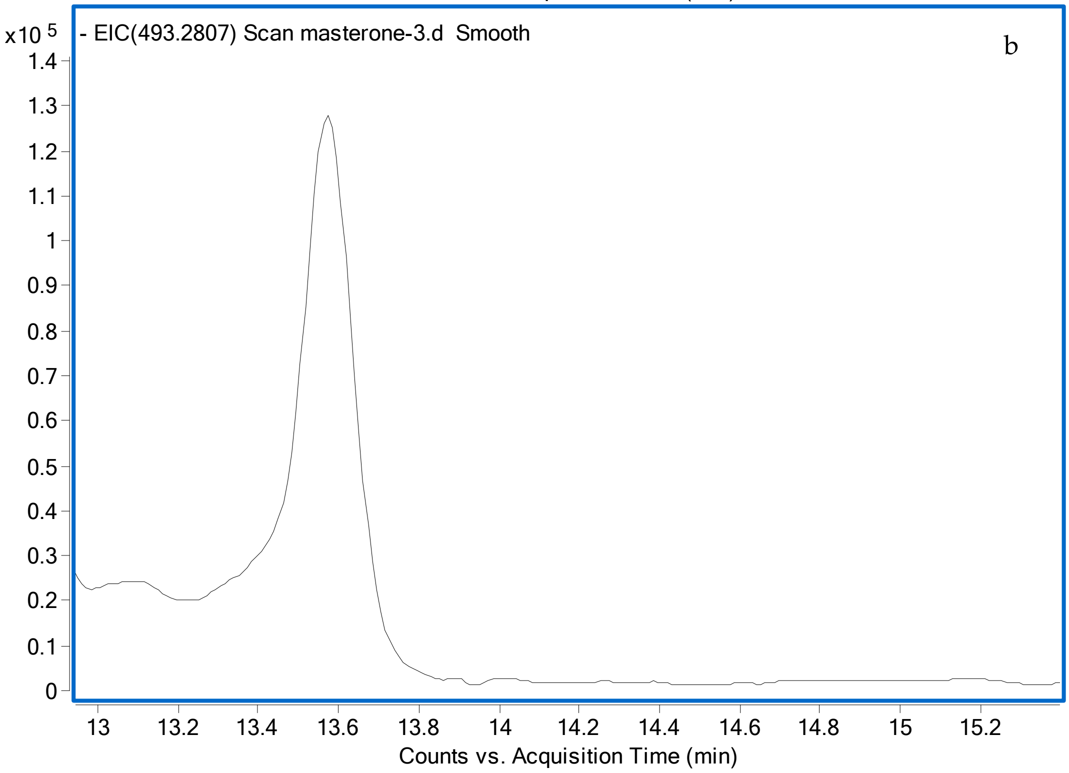

Figure S1. *Cont.*

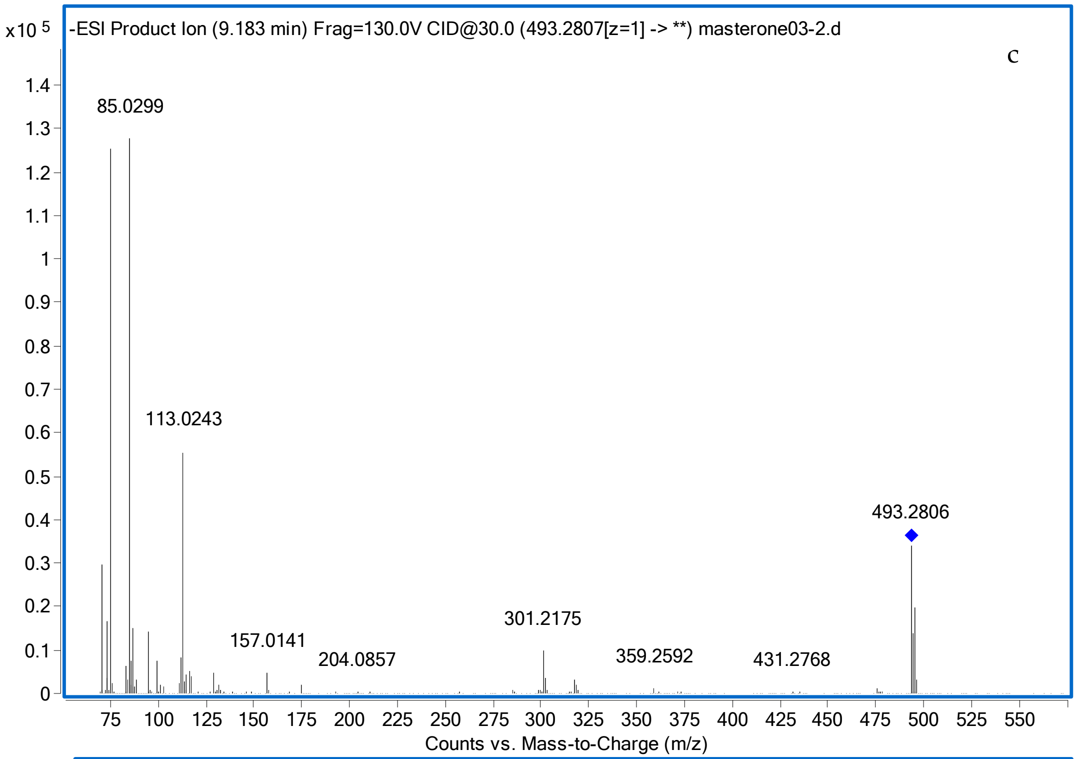

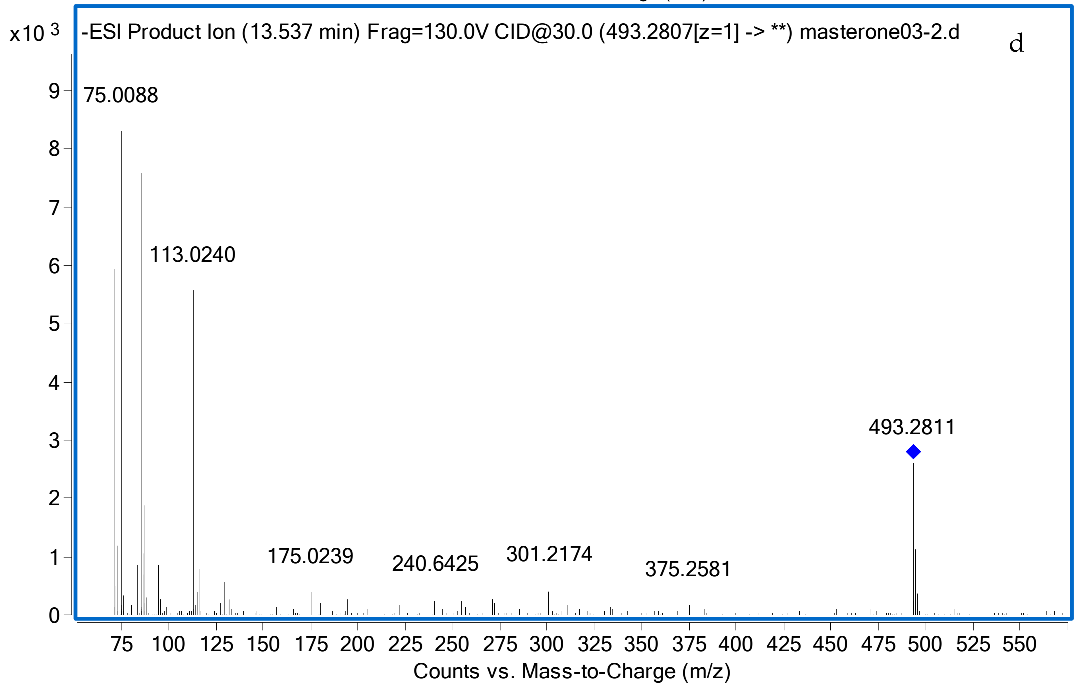

Figure S1. The EIC and product ion scan chromatograms of G1 (**a**,**c**) and G2 (**b**,**d**) in LC-QTOF-MS negative mode.

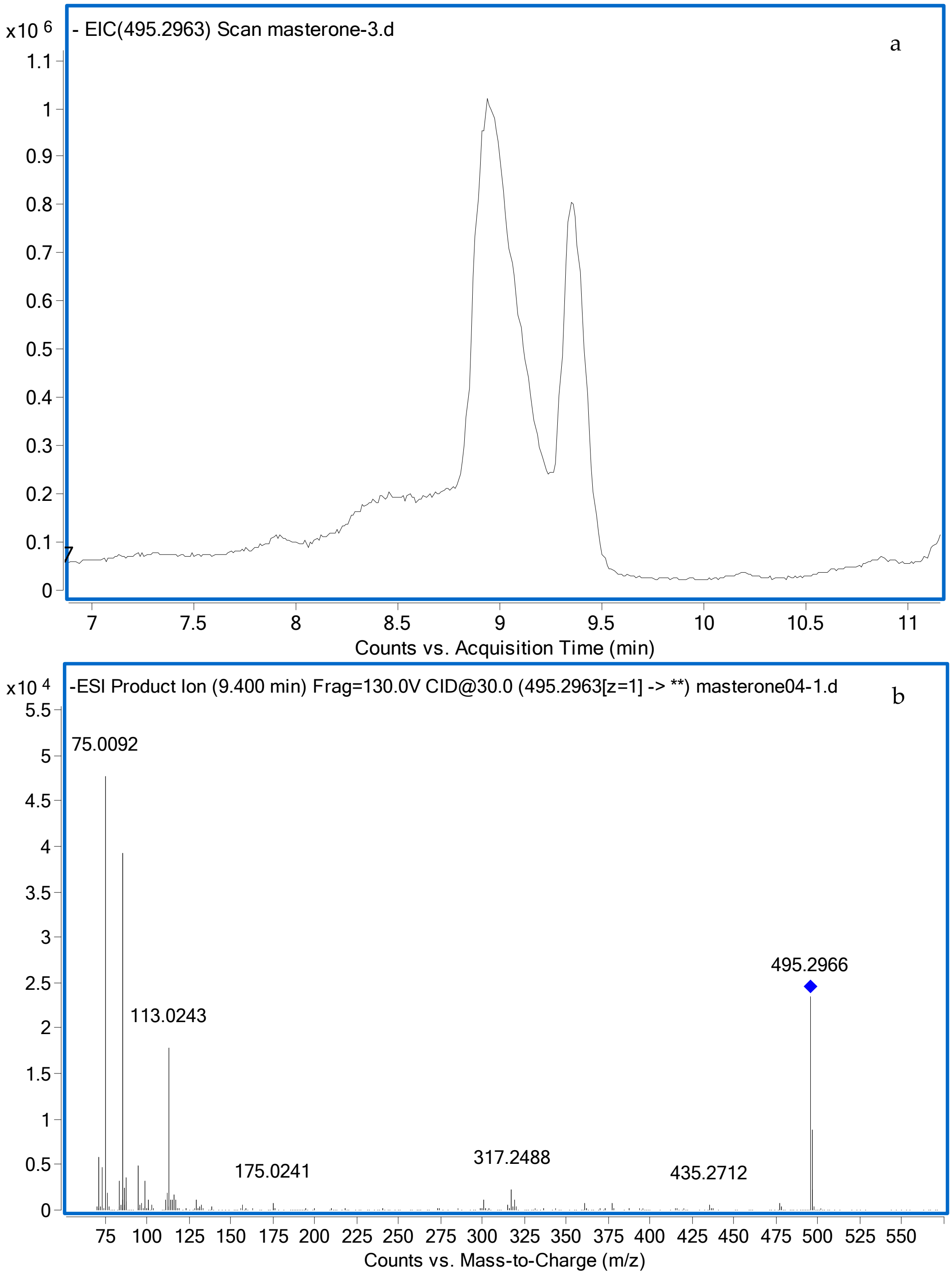

Figure S2. The EIC (**a**) and product ion scan chromatogram (**b**) of G3 (RT: 9.4 min) in LC-QTOF-MS negative mode.

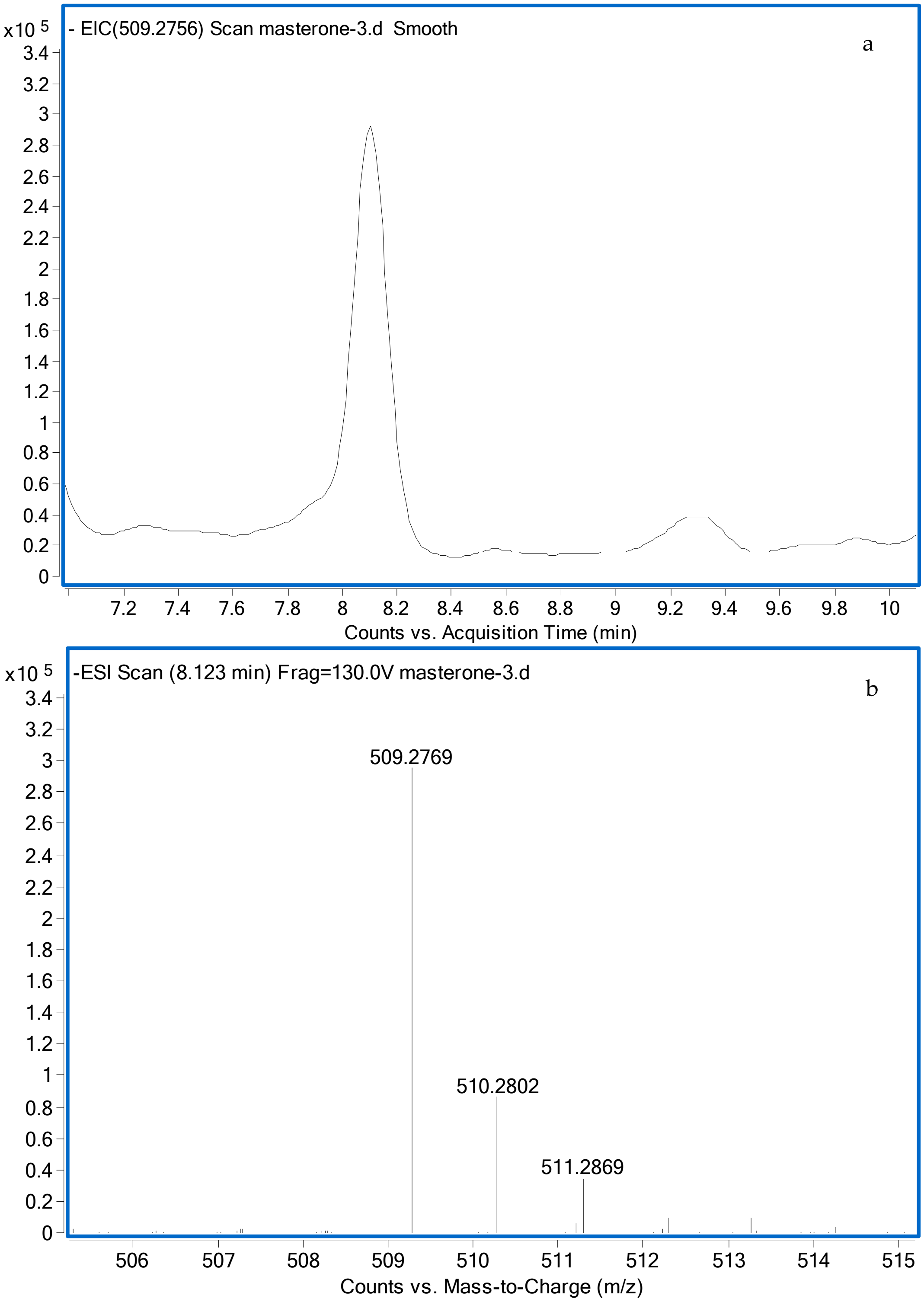

Figure S3. The EIC (**a**) and product ion scan chromatogram (**b**) of G4 in LC-QTOF-MS negative mode.

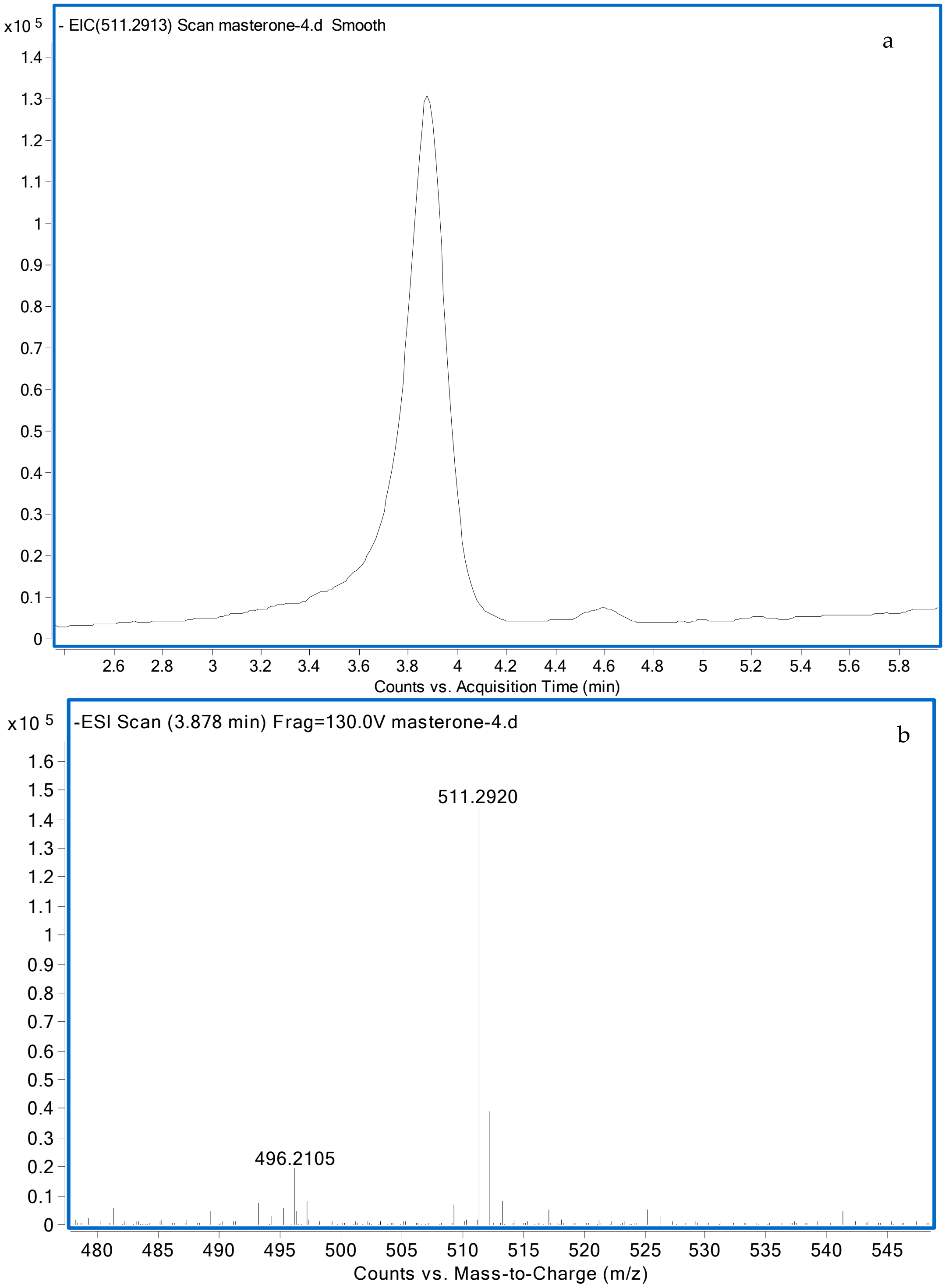

Figure S4. The EIC (**a**) and full scan chromatogram (**b**) of G5 in LC-QTOF-MS negative mode.

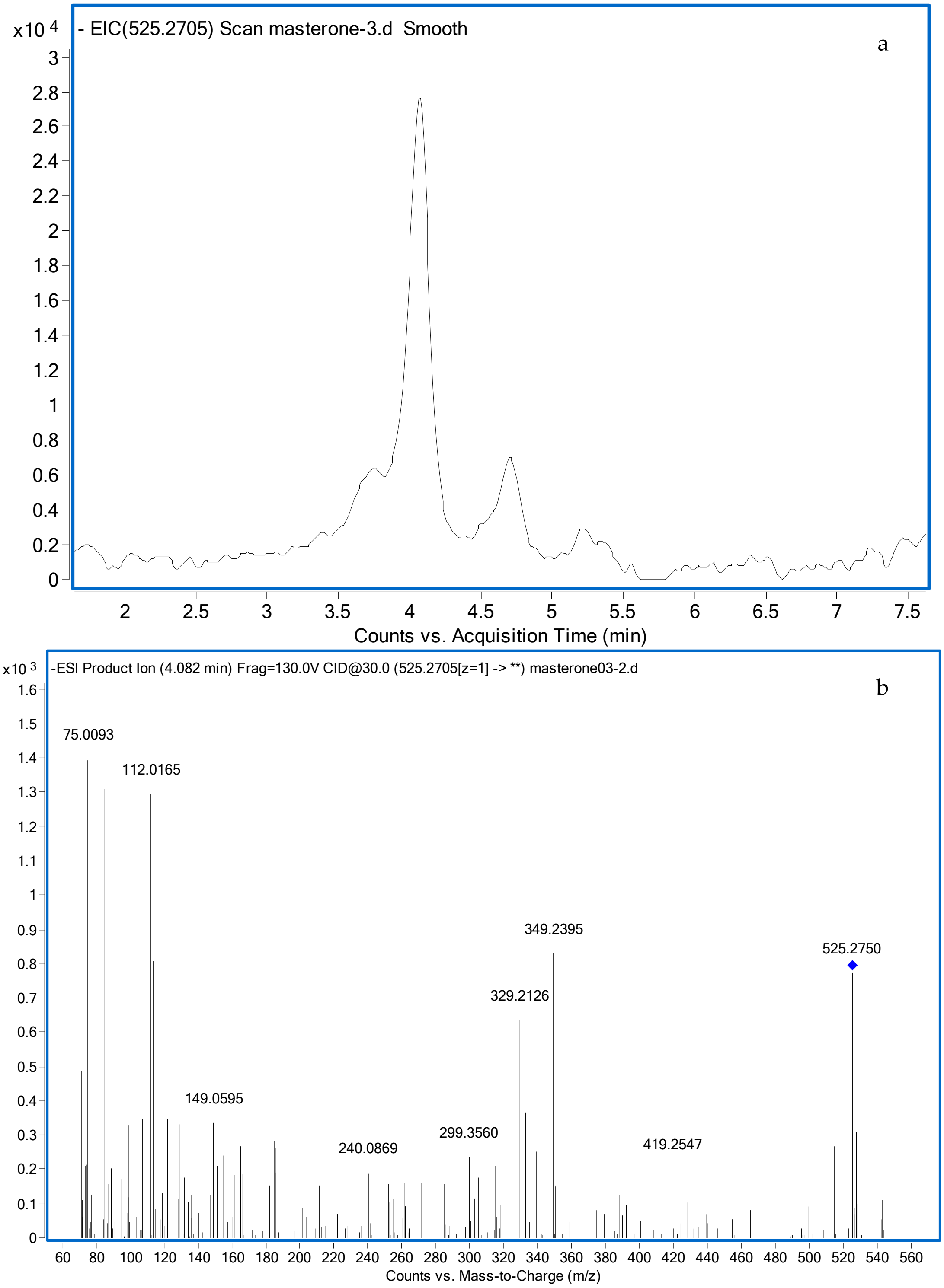

Figure S5. The EIC (**a**) and product ion chromatogram (**b**) of G6 in LC-QTOF-MS negative mode.

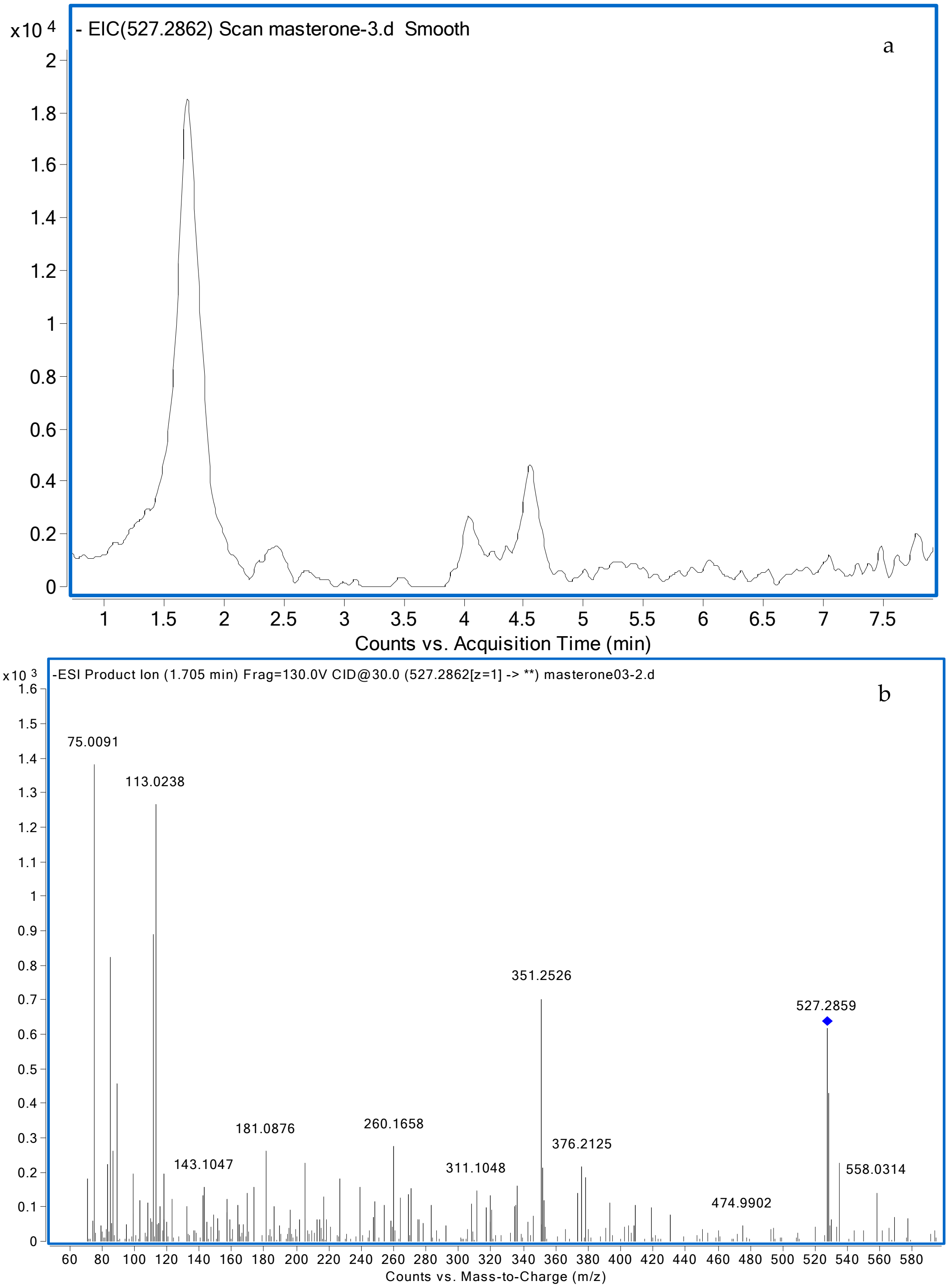

Figure S6. The EIC (**a**) and product ion chromatogram (**b**) of G7 in LC-QTOF-MS negative mode.

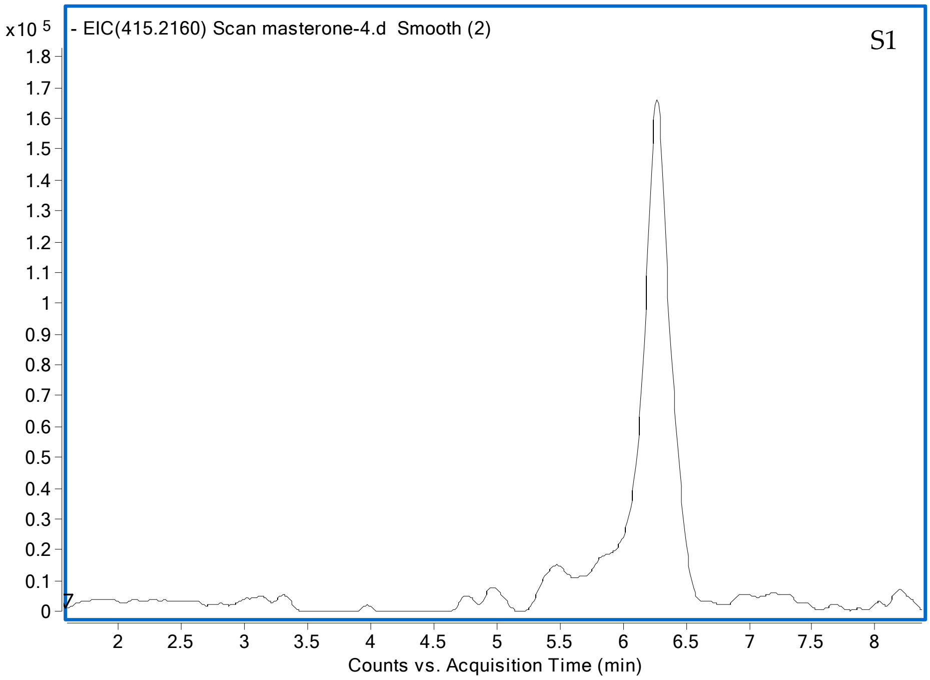

Figure S7. The EIC of S1 in LC-QTOF-MS negative mode.

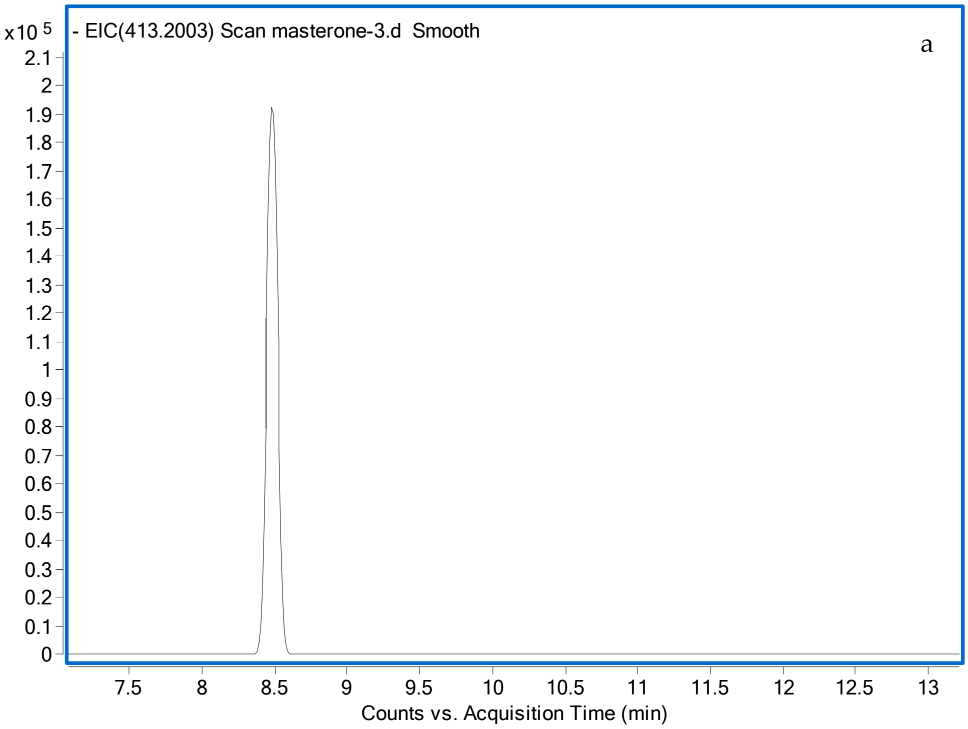

Figure S8. *Cont.*

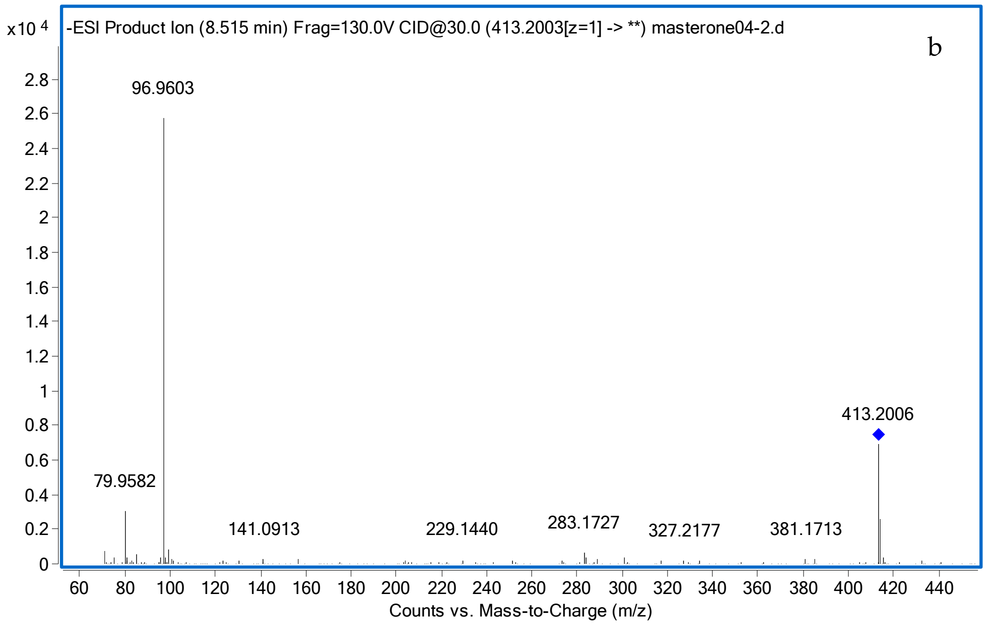

Figure S8. The EIC (**a**) and product ion chromatogram (**b**) of S2 in LC-QTOF-MS negative mode.

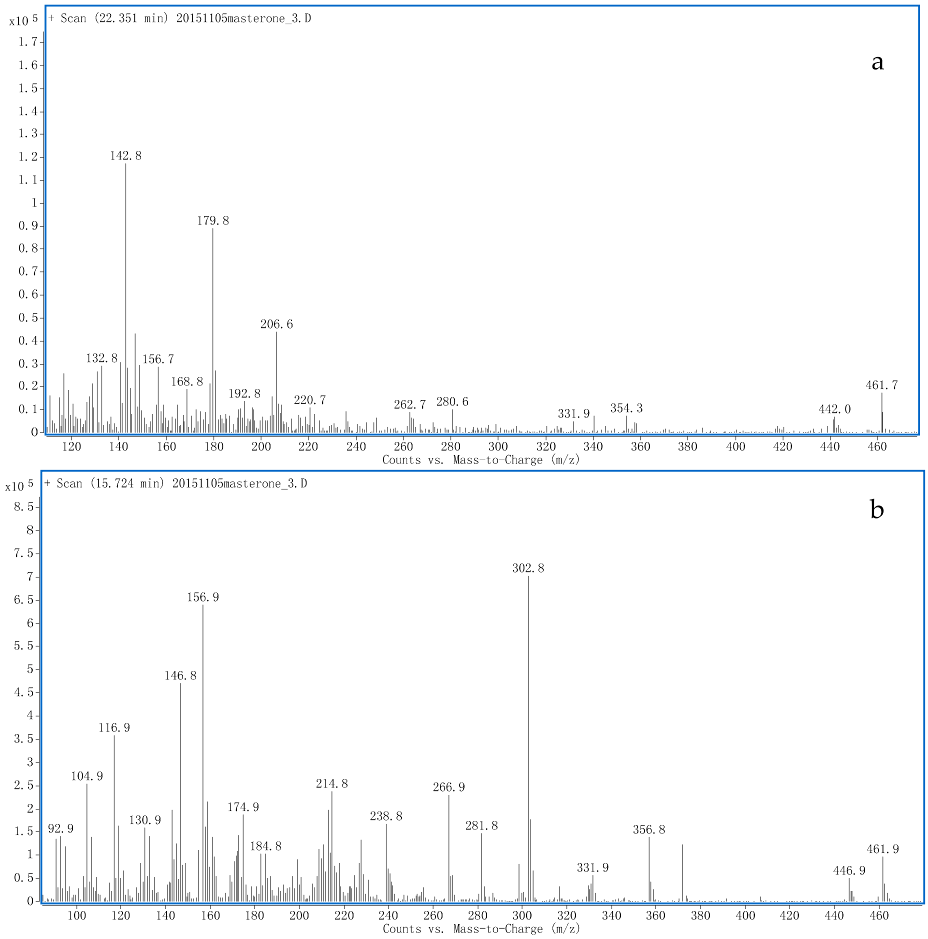

Figure S9. *Cont.*

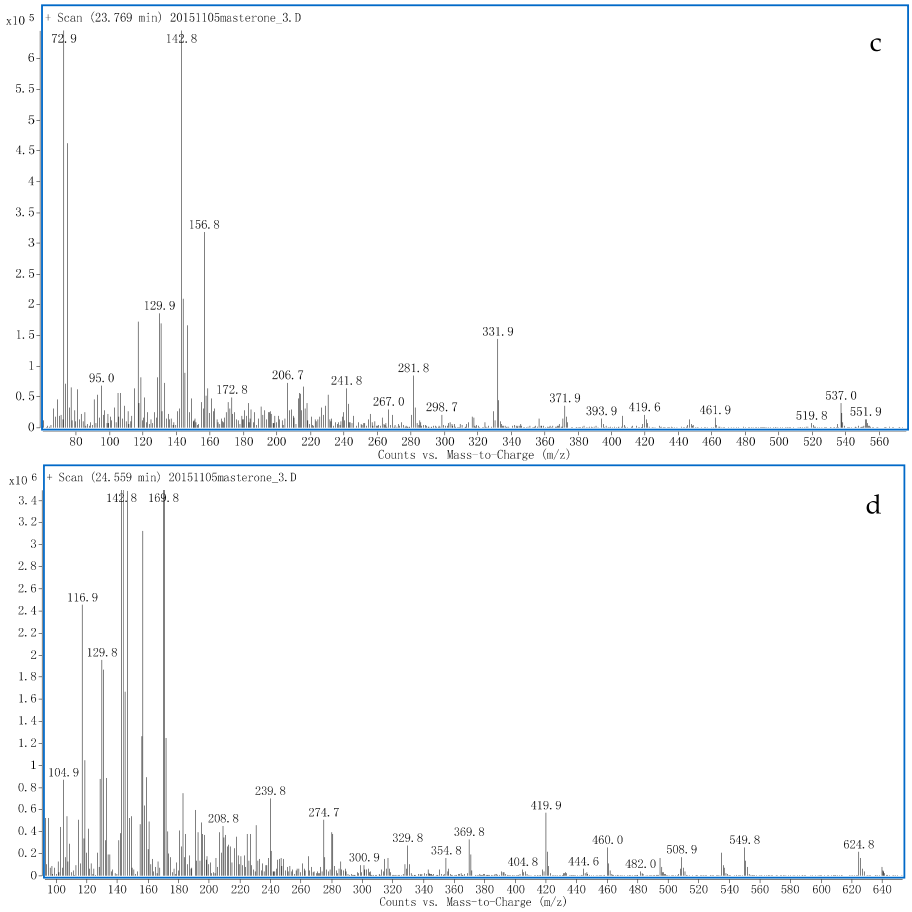

Figure S9. The Electron ionization mass spectra of TMS derivatives of M1(**a**), M2(**b**), M3(**c**), M5(**d**).
